# Characterizing herpes simplex virus type 1 and type 2 seroprevalence declines and epidemiological association in the United States

**DOI:** 10.1371/journal.pone.0214151

**Published:** 2019-06-06

**Authors:** Hiam Chemaitelly, Nico Nagelkerke, Ryosuke Omori, Laith J. Abu-Raddad

**Affiliations:** 1 Infectious Disease Epidemiology Group, Weill Cornell Medicine-Qatar, Cornell University, Qatar Foundation—Education City, Doha, Qatar; 2 Division of Bioinformatics, Research Center for Zoonosis Control, Hokkaido University, Sapporo, Hokkaido, Japan; 3 Department of Healthcare Policy & Research, Weill Cornell Medicine, Cornell University, Ithaca, New York, United States of America; 4 College of Health and Life Sciences, Hamad bin Khalifa University, Doha, Qatar; Rosalind Franklin University of Medicine and Science, UNITED STATES

## Abstract

**Objective:**

Assessing the epidemiological association between herpes simplex virus type 1 (HSV-1) and type 2 (HSV-2) infections in the United States, and characterizing the trends in the standardized HSV-1 and HSV-2 antibody prevalences (seroprevalences), 1999–2016.

**Methods:**

Source of data was the cross-sectional and nationally-representative biennial surveys of the National Health and Nutrition Examination Survey (NHANES). All nine NHANES rounds for 1999–2016 were included in analysis. Datasets of these rounds were combined and analyzed accounting for survey design and applying weighting procedures. Logistic regressions were used to identify associations with seropositivity. Sensitivity analyses were conducted.

**Results:**

Odds of HSV-1 infection declined by 2.84% (95% CI: 1.70%-4.00%) annually among men, and by 2.22% (95% CI: 1.23%-3.21%) among women. Declines were highest at younger ages. Odds of HSV-2 infection declined by 2.23% (95% CI: 0.71%-3.82%) annually among men, and by 2.89% (95% CI: 1.57%-4.28%) among women. Odds ratio of the association between HSV-2 and HSV-1 seropositivity was 0.71 (95% CI: 0.60–0.84) for men and 0.81 (95% CI: 0.72–0.91) for women, after adjustment for age, ethnicity, and year.

**Conclusion:**

HSV-1 and HSV-2 seroprevalences showed a strong declining trend for at least two decades, for both sexes and for the different ethnicities, possibly reflecting improvements in hygiene and living conditions (for HSV-1), and safer sexual behavior (for HSV-2). HSV-1 seroprevalence declines are most pronounced among young individuals. There is evidence for cross protection between the two infections, suggestive of HSV-1 seropositivity being partially protective against HSV-2 infection.

## Introduction

Herpes simplex virus type 1 (HSV-1) and type 2 (HSV-2) are viruses that establish life-long infections in humans [1]. The major route of transmission for HSV-1 is oral, although (oral) sexual transmission is increasingly common in Western countries and Asia [2–4]. Sexual transmission is the main route of transmission for HSV-2 [5]. Infection by these viruses is often latent and asymptomatic, with frequent reactivations and occasional intermittent symptomatic episodes [6, 7]. Infection can be ascertained by antibody tests (“seroprevalence”) [8]. HSV-1/2 vaccine development is a focus of ongoing international effort [9, 10].

The epidemiologies of HSV-1 and HSV-2 infections are important because of the clinical and psychosocial disease burden and inconvenience these infections can cause [2, 11]. In addition, HSV-2 has been implicated as a cofactor in HIV transmission [12, 13], although recent work has cast doubts on this association [14]. Nevertheless, if true, its control, say through a vaccine, may lead to substantial reductions in HIV transmission [15, 16].

HSV-1 and HSV-2 infections are also indicators of behaviors that facilitate the spread of other infections. Notably, HSV-2 seroprevalence (and seroincidence) reflects sexual risk behavior, and may serve as a marker for potential HIV spread [17–19]. As these infections are viral, they are not influenced by treatment patterns, in contrast to curable bacterial infections. However, as they persist for life, changes in transmission dynamics affect their seroprevalences with variable delays.

In the United States, HSV-2 seroprevalence increased between the 1970s and early 1990s, and was projected to increase to 39% among men and 49% among women aged 15–39 by 2025 [20]. Contrary to these projections, using data from the National Health and Nutrition Examination Survey (NHANES), Xu *et al*. found a 19% decline in seroprevalence over the period 1999–2004, relative to 1988–1994 [21]. More recently, a data brief, using more recent rounds of NHANES data, suggested persistent declines in seroprevalence [22]. This may suggest that either the earlier high seroprevalence may have been a transient phenomenon, or that the projected increase had been delayed, or avoided, perhaps due to safer sexual behavior following concerns about HIV. With the advent of HIV antiretroviral therapy and pre-exposure prophylaxis [23, 24], however, such concerns might have diminished, leading potentially to a reversion in HSV-2 seroprevalence trajectory. We aimed in this article to statistically investigate and characterize the recent trends in HSV-2 seroprevalence over the period 1999–2016.

HSV-1 is commonly acquired in childhood [1], but changes in hygiene and socio-economic conditions reduced exposure in childhood in Western and Asian countries [1, 3, 21]. Young individuals are increasingly entering the sexually active phase without previous HSV-1 exposure, thus being at risk of HSV-1 genital acquisition and genital herpes [2, 4, 25]. HSV-1 could be overtaking HSV-2 as the main cause of first episode of genital herpes in the United States and elsewhere [2, 4, 25]. The extent to which HSV-1 seroprevalence is declining in childhood in the United States, exposing adults to increasing HSV-1 genital herpes, remains not well-understood. Thus, we further aimed here to statistically investigate and characterize the recent trends in HSV-1 seroprevalence over the period 1999–2016.

HSV-1 and HSV-2 are closely-related antigenically raising a question about the potential for an epidemiological interaction between the two infections. Specifically, since HSV-1 is normally acquired in childhood [1, 3], could prior HSV-1 infection be protective against subsequent HSV-2 infection, which normally occurs after sexual debut [1, 5]? Of notice that the two viruses typically infect two different sites, oral versus genital [2, 26]—two different biological niches, with a variation in immune response [27], that may reduce any immunological cross protection [28]. The two viruses could also have evolved to escape any cross protection to sustain circulation in human populations, particularly for HSV-2, which is normally acquired after HSV-1 [28]. Therefore, we further aimed to assess the evidence for an epidemiological interaction between these two infections using the NHANES database—the world’s foremost nationally-representative database of population-based repeated surveys for both, HSV-1 and HSV-2 infections.

## Methods

NHANES are nationally-representative (for the non-institutionalized United States population) probability-based surveys. For sampling, the country is divided into geographic areas (mostly single counties), called "primary sampling units (PSUs)". Strata within PSUs are divided into neighborhoods from which households are randomly selected. Eligible inhabitants of those households are interviewed and a subsample tested for the presence of glycoprotein specific HSV-1 (designated gG-1) and HSV-2 (designated gG-2) in sera using highly sensitive and specific solid-phase enzymatic type-specific immunodot assays [29, 30]. As some demographic groups are oversampled, observations are weighted to obtain truly representative samples.

We used the publicly-available continuous NHANES data from a total of nine biennial surveys (“waves” or “rounds”) extending over the period 1999–2016 [31]. All surveys conducted during this period followed a standardized methodology, both analytically and in laboratory procedures. HSV-1 serological test results were available for ages 14–49, and HSV-2 tests for ages 18–49. Rounds, here, will be denoted by their first (calendar) year (e.g. 1999–2000 = “1999”).

Datasets were combined and analyzed using STATA 13.0 [32], taking into account surveys’ design and applying recommended weighting procedures (using PSUs, strata, and sampling weights). NHANES uses separate strata numbers for different rounds so that the combined data is effectively a survey from a superpopulation that consists of nine “replicas” of the United States population, one from each round (S1 Dataset). Graphical illustrations were made in R version 3.5.1 [33].

Logistic regression was used to identify associations with seropositivity. All regressions were stratified by sex. Age (years) was treated as a categorical variable, and calendar year as a continuous variable, after verifying that there were no major non-linear trends. Thus, annual declines in odds of infection (seroprevalence of infection/seroprevalence of non-infection) were obtained from logistic analysis with calendar year (NHANES round) being a continuous variable, and age being a categorical variable. This method of analysis (as opposed to prevalence ratios), was deemed optimal for statistical [34] and scientific reasons relating to the specific research questions of this study, and for better reflection of incidence ratios (or forces of infection).

Further analyses were also carried out, mostly to scrutinize results through sensitivity analyses. First, we explored whether there were heterogeneities in trend among ethnic groups (distinguishing Mexican, Other Hispanic, White, Black, and Other ethnicities) within the United States population. We further examined ethnicity’s interaction with calendar year. An additional interaction that was explored was that between age (here as a continuous variable) and calendar year. This interaction was examined to explore differences in trend among different age groups. Last but not least, in order to explore the association between HSV-1 and HSV-2 infections, we carried out a logistic regression for HSV-2 infection in which HSV-1 infection was included as an additional covariable.

To obtain standardized seroprevalences by year and sex from age-sex-year specific population seroprevalences, we applied direct standardization, using the United States 2010 population as a reference [35] (its 14–49 and 18–49 populations for HSV-1 and HSV-2, respectively). To estimate these age-sex-year specific population seroprevalences, we carried out (survey) logistic regression with all interactions between age and year as independent variables (all as categorical variables), for each sex, thereby adjusting the survey seroprevalences for the NHANES weights and other design aspects. We used loess (locally weighted polynomial regression) to calculate interpolating curves.

## Results

**Fig 1:**
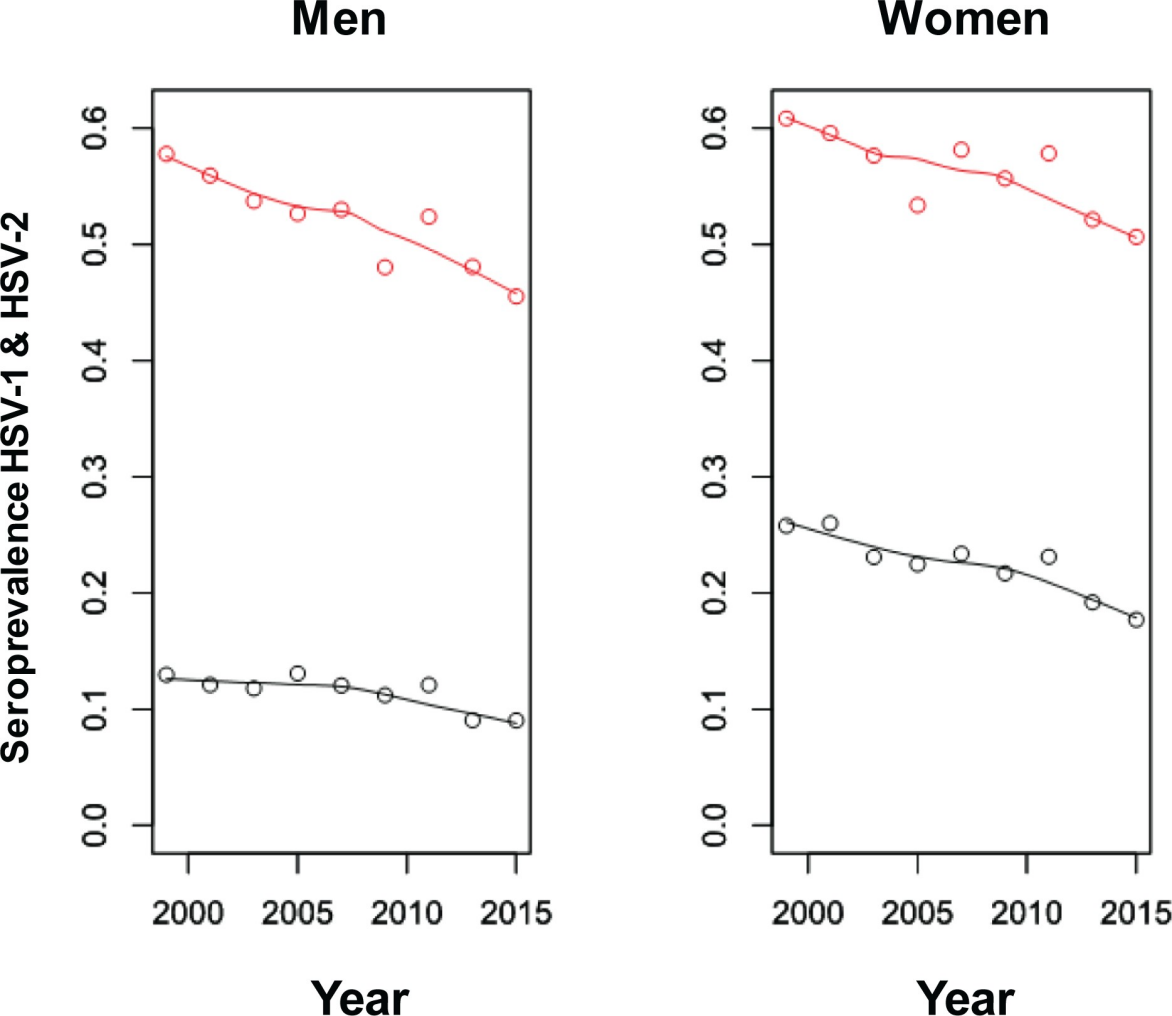
Trends in seroprevalence of HSV-1 (red) and HSV-2 (black) infections in the United States, 1999–2015. Seroprevalences were standardized with respect to the United States 2010 population; ages 14–49 for HSV-1 and 18–49 for HSV-2. Interpolating curves were estimated using loess.

**Fig 2:**
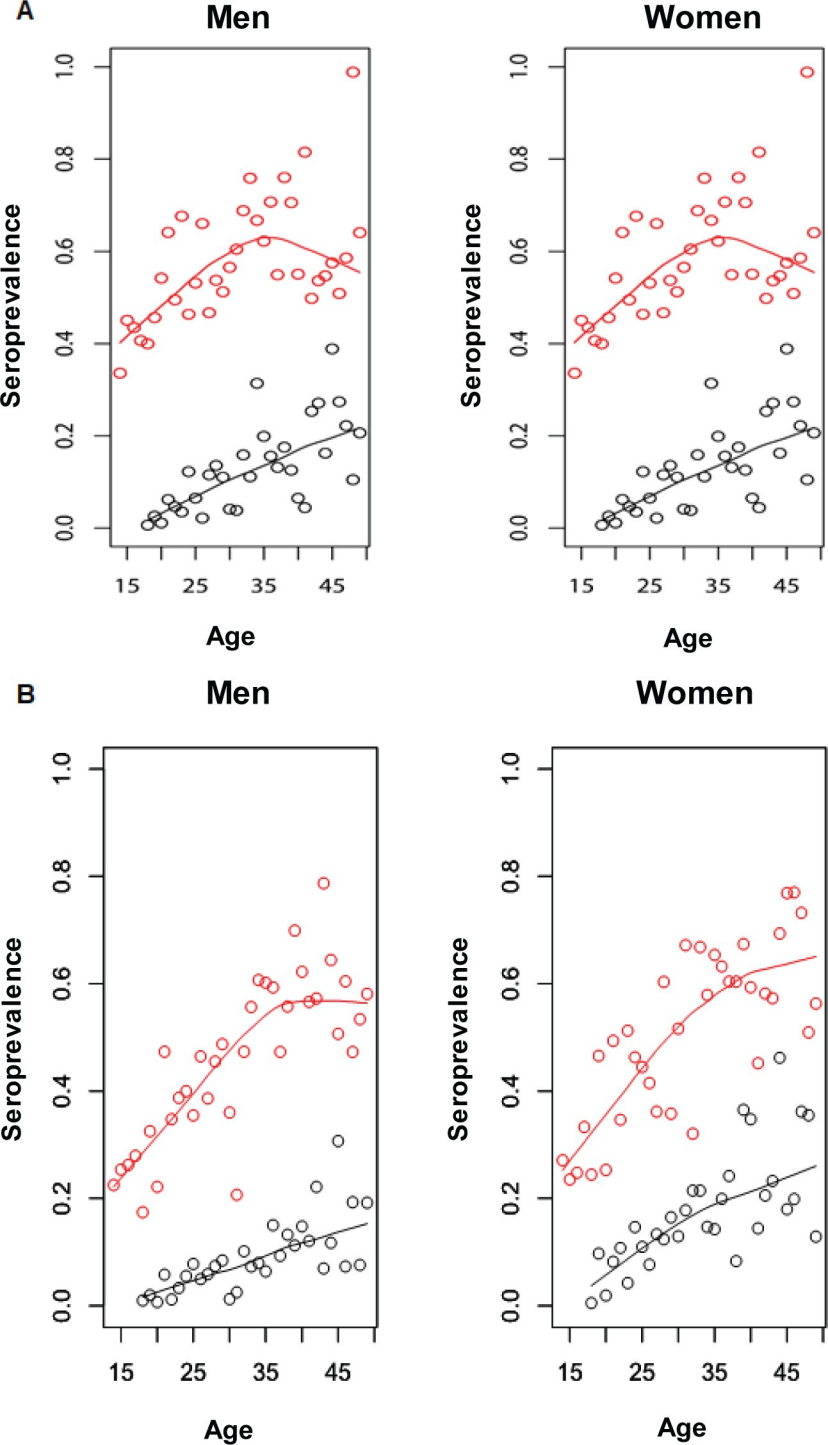
Estimated age-specific HSV-1 (red) and HSV-2 (black) seroprevalence in the United States. A) Estimates using 1999–2000 data. B) Estimates using 2015–2016 data. Estimates were derived from survey logistic regression (points), as well as loess smoothed estimates (curves).

Fig 1 shows the trends for the standardized seroprevalences of HSV-1 and HSV-2 infections, as well as the loess smoothed estimates. HSV-1 and HSV-2 seroprevalences, stratified by age and sex, over the period 1999–2016, can be found in S1 Table. Fig 2A and 2B show the estimated age-specific seroprevalences of HSV-1 and HSV-2 infections, for men and women, in the years 1999–2000 and 2015–2016, respectively, as well as their loess smoothed estimates.

### HSV-1 seroprevalence

There were an (unweighted) total of 7144 and 6713 HSV-1 negative tests, and 8761 and 10330 positive tests for men and women, aged 14–49, respectively. Using (survey) logistic regression, the odds of HSV-1 infection declined by 2.84% (95% CI: 1.70%-4.00%) and 2.22% (95% CI: 1.23%-3.21%) per year among men and women, respectively. While there were large differences in HSV-1 seroprevalence by ethnicity, the relative declines in seroprevalence were rather similar among the different ethnicities (S1 Fig).

Through sensitivity analysis, there was, however, a highly significant interaction between age (as a continuous variable) and year, with the strongest declines in HSV-1 seroprevalence in the younger age groups for both men and women—the declines decreased in strength (odds) by 0.0013, i.e. 0.13% (95% CI: 0.05–0.21%) per year of age among men, and by 0.18% (95% CI: 0.10–0.25%) per year of age among women.

### HSV-2 seroprevalence

There were a total of 10657 and 10316 HSV-2 negative tests, and 1631 and 3311 positive tests for men and women, respectively—much lower overall seroprevalence than for HSV-1. The odds of HSV-2 infection declined by 2.23% (95% CI: 0.71%-3.82%) and 2.89% (95% CI: 1.57%-4.28%) per year among men and women, respectively. While there were large differences in HSV-2 seroprevalence by ethnicity, the relative declines in seroprevalence were overall similar among the different ethnicities (S2 Fig). Through sensitivity analysis, there was no significant interaction between age and year.

### Association between HSV-1 and HSV-2 infections

In the additional logistic regression for HSV-2 infection, in which HSV-1 infection was included as an independent covariable, the odds ratio of HSV-2 infection with HSV-1 seropositivity was 0.71 (95% CI: 0.60–0.84; p≤0.001) for men, and 0.81 (95% CI: 0.72–0.91; p≤0.001) for women, after adjustment for age, ethnicity, and year.

## Discussion

Recent trends in HSV-1 and HSV-2 seroprevalences in the United States were assessed using the NHANES database. Findings showed continuing declines in both HSV-1 and HSV-2 seroprevalences up to the present (Fig 1), consistent with earlier observed declines [21, 36, 37], and confirming a recent summary report on these data [22]. These declines were strong regardless of sex or ethnicity (Fig 1 and S1 and S2 Figs), but for HSV-1, were more pronounced for the younger age cohorts. Women showed consistently higher HSV-2 seroprevalence than men, possibly due to differences in biological susceptibility [1, 38–40]. Importantly, there was evidence for a negative association between HSV-1 and HSV-2 infections, suggestive of some immunity cross-protection.

The continuing declines in HSV-1 seroprevalence, notably in childhood, may reflect progressive improvements in hygiene and living conditions, and demonstrate how HSV-1 seroprevalence has deviated from its historical pattern of nearly universal infection, still observed in other parts of the globe, where no declines have been documented [3, 41, 42]. While a decline in infection seroprevalence is a positive development, implying decreases in oral herpes and more serious sequelae [2], it is of concern, as young individuals are increasingly unprotected against HSV-1 genital acquisition (with their steadily declining seroprevalence before sexual debut). The role of HSV-1 in genital herpes will probably increase, as HSV-1 seroprevalence declines further in the younger age cohorts.

The continuing declines in HSV-2 seroprevalence affirm that the earlier observed declines [21] were not just a transient phenomenon, but may reflect consistently lower sexual risk behavior, including changes in sexual networking, or use of barrier contraceptive methods. The high observed seroprevalence of late 1970s to early 1990s [20] may have been exceptional, and the consequence of riskier sexual behavior during the sexual revolution of the preceding two decades. Trends in HSV-2 seroprevalence in other parts of the world are yet to be ascertained with confidence [39].

Remarkably, rates of human papillomavirus infection, another viral STI, seem also to have declined in recent decades in the United States [43]. Based on suggestive evidence, these declines may not have been solely due to HPV vaccination [44, 45], thus supporting declines in sexual risk behavior as an explanation. Self-reported behavior data further indicates lower levels of sexual behavior, particularly among young individuals. In the National Survey of Family Growth [46] of 2006–2008 compared to that of 2002, the percentage of men and women aged 15–24 who never had a sexual contact increased from 22% (men and women) to 27% (men) and 29% (women). The declines could be further due to changing sexual-mixing patterns, which can significantly affect the spread of STIs [47, 48]. Whether age-mixing has changed over the past decades, say due to rising college education with approximately (currently) equal men and women participation, remains to be explored.

Current evidence on a possible epidemiological interaction between HSV-1 and HSV-2 infections is conflicting [28]. Pooled findings of prospective studies suggest a lower risk of HSV-2 infection with prior HSV-1 infection [28]; but pooled findings of cross-sectional studies suggest a higher risk of HSV-2 infection with HSV-1 infection in low sexual risk populations and in Europe, but no association in high sexual risk populations and in the United States [28]. Our results suggest some cross-protection. Most likely, assuming that HSV-1 infection precedes HSV-2 infection [3, 17, 41], a lower risk of HSV-2 infection after HSV-1 infection [49], just as the pooled finding of prospective studies. Of notice, a recent study on a merged multi-national database of seroprevalence studies among men, also suggested that HSV-1 infection provided protection against HSV-2 acquisition, with an adjusted odds ratio of 0.51 (95% CI 0.30–0.87; p-value = 0.013) (Nasrallah G.K., Personal communication), similar to the present study result. It would be useful to explore further this negative association and its underlying causes within the complex and delicate web of innate signaling pathways and adaptive immune responses against HSV infections [49].

Our study was concerned with seroprevalence, a measure of lifetime exposure to the infection, and thus may not necessarily reflect actual current trends in transmission dynamics. The analyzed data are based on repeated rounds of surveys, and therefore, no seroconversions were identifiable, allowing only indirect conclusions about incidence. NHANES rounds did not include the institutionalized populations of the United States, such as the incarcerated population, and thus, our results may not apply to the institutionalized populations. The association between HSV-1 and HSV-2 infections was assessed using cross-sectional data, and thus we cannot strictly infer the temporal sequence of cause and effect.

In conclusion, HSV-1 and HSV-2 epidemiologies continue to evolve in the United States, with the seroprevalence of both infections progressively declining for both sexes, and for the different ethnicities. The declines in HSV-1 seroprevalence are more pronounced for the younger age cohorts. Younger cohorts are increasingly acquiring the infection for the first time genitally after sexual debut, as opposed to orally during childhood, thus pointing towards an increasing role for HSV-1 in genital herpes. Importantly, there was evidence for an HSV-1/2 epidemiological interaction, suggestive of HSV-1 infection being partially protective against HSV-2 infection.

## Supporting information

S1 Dataset Dataset for HSV-1 and HSV-2 serological markers using the National Health and Nutrition Examination Survey continuous rounds for the period 1999–2016. (XLS)

S1 Table HSV-1 and HSV-2 seroprevalence in the United States stratified by age and sex, based on the National Health and Nutrition Examination Survey data for the period 1999–2016. (DOCX)

S1 Fig Trends in seroprevalence of HSV-1 infection in the United States stratified by sex and ethnicity, 1999–2015. Seroprevalence was standardized with respect to the United States 2010 population (ages 14–49). Interpolating curves were estimated using loess.
(TIF)

S2 Fig Trends in seroprevalence of HSV-2 infection in the United States stratified by sex and ethnicity, 1999–2015. Seroprevalence was standardized with respect to the United States 2010 population (ages 18–49). Interpolating curves were estimated using loess.
(TIF)
