# Supplementary material for: Characterizing herpes simplex virus type 1 and type 2 seroprevalence declines and epidemiological association in the United States
Source: PLoS One. 2019 Jun 6;14(6):e0214151. doi: 10.1371/journal.pone.0214151 (PMC6553692; doi:10.1371/journal.pone.0214151)
Supplement: S1 Table — (DOCX) [file pone.0214151.s002.docx]

**S1 Table.** **HSV-1 and HSV-2 seroprevalence in the United States stratified by age and sex, based on the National Health and Nutrition Examination Survey data for the period 1999-2016.**

| **NHANES round** | **HSV-1 seroprevalence** | | | | | | **HSV-2 seroprevalence** | | | | | |
| --- | --- | --- | --- | --- | --- | --- | --- | --- | --- | --- | --- | --- |
|  | **Men** |  |  | **Women** |  |  | **Men** |  |  | **Women** |  |  |
|  | **N tested** | **N positive** | **%^*^** | **N tested** | **N positive** | **%^*^** | **N tested** | **N positive** | **%^*^** | **N tested** | **N positive** | **%^*^** |
| **Age 14-19 years** |  |  |  |  |  |  |  |  |  |  |  |  |
| 1999-00 | 769 | 407 | 41.6 | 756 | 424 | 45.1 | 243 | 5 | 1.7 | 253 | 17 | 5.1 |
| 2001-02 | 810 | 379 | 36.6 | 797 | 408 | 40.7 | 270 | 5 | 1.4 | 239 | 17 | 6.3 |
| 2003-04 | 783 | 344 | 32.3 | 729 | 352 | 38.6 | 243 | 6 | 1.1 | 265 | 16 | 3.0 |
| 2005-06 | 735 | 268 | 24.8 | 735 | 316 | 33.1 | 256 | 4 | 1.2 | 251 | 8 | 1.7 |
| 2007-08 | 437 | 156 | 29.0 | 378 | 164 | 36.9 | 143 | 2 | 1.0 | 110 | 11 | 8.4 |
| 2009-10 | 488 | 183 | 27.5 | 405 | 168 | 29.4 | 148 | 1 | 0.3 | 126 | 6 | 3.3 |
| 2011-12 | 412 | 155 | 33.8 | 386 | 162 | 32.3 | 135 | 2 | 0.4 | 132 | 3 | 1.6 |
| 2013-14 | 451 | 142 | 26.1 | 468 | 171 | 29.8 | 134 | 0 | 0 | 160 | 6 | 3.5 |
| 2015-16 | 419 | 136 | 25.4 | 391 | 133 | 28.8 | 127 | 2 | 1.1 | 107 | 6 | 4.2 |
| **Age 20-24 years** |  |  |  |  |  |  |  |  |  |  |  |  |
| 1999-00 | 149 | 94 | 57.2 | 222 | 160 | 62.0 | 147 | 8 | 3.2 | 223 | 23 | 8.8 |
| 2001-02 | 197 | 108 | 49.6 | 273 | 166 | 55.1 | 196 | 14 | 5.8 | 274 | 50 | 13.6 |
| 2003-04 | 195 | 102 | 48.4 | 228 | 133 | 53.1 | 195 | 9 | 2.7 | 228 | 36 | 11.4 |
| 2005-06 | 191 | 104 | 50.0 | 274 | 163 | 50.0 | 190 | 16 | 7.1 | 273 | 31 | 8.0 |
| 2007-08 | 187 | 86 | 41.8 | 212 | 124 | 48.8 | 187 | 16 | 6.7 | 211 | 29 | 12.6 |
| 2009-10 | 236 | 105 | 34.9 | 261 | 136 | 44.8 | 236 | 6 | 1.8 | 261 | 33 | 11.7 |
| 2011-12 | 251 | 109 | 39.1 | 228 | 118 | 45.7 | 251 | 8 | 3.8 | 229 | 27 | 9.8 |
| 2013-14 | 234 | 94 | 35.5 | 230 | 107 | 40.5 | 233 | 5 | 1.3 | 228 | 20 | 6.6 |
| 2015-16 | 183 | 76 | 35.6 | 197 | 97 | 42.2 | 183 | 8 | 2.9 | 197 | 20 | 7.7 |
| **Age 25-29 years** |  |  |  |  |  |  |  |  |  |  |  |  |
| 1999-00 | 149 | 89 | 54.4 | 199 | 135 | 62.9 | 149 | 15 | 9.2 | 199 | 40 | 16.6 |
| 2001-02 | 164 | 96 | 48.7 | 247 | 166 | 60.7 | 164 | 17 | 8.0 | 248 | 53 | 21.5 |
| 2003-04 | 188 | 106 | 52.3 | 193 | 106 | 50.6 | 187 | 13 | 5.4 | 192 | 49 | 22.0 |
| 2005-06 | 180 | 113 | 53.2 | 278 | 170 | 53.3 | 180 | 12 | 5.0 | 278 | 65 | 21.9 |
| 2007-08 | 199 | 119 | 53.6 | 188 | 122 | 57.2 | 199 | 22 | 7.5 | 188 | 42 | 15.8 |
| 2009-10 | 203 | 109 | 47.2 | 248 | 161 | 59.2 | 203 | 22 | 8.1 | 248 | 45 | 13.5 |
| 2011-12 | 190 | 98 | 48.4 | 203 | 113 | 51.8 | 190 | 17 | 5.8 | 202 | 35 | 13.9 |
| 2013-14 | 200 | 87 | 41.6 | 211 | 120 | 50.1 | 200 | 16 | 5.5 | 208 | 37 | 14.2 |
| 2015-16 | 219 | 112 | 42.7 | 259 | 139 | 43.8 | 219 | 17 | 6.8 | 259 | 39 | 12.0 |
| **Age 30-34 years** |  |  |  |  |  |  |  |  |  |  |  |  |
| 1999-00 | 155 | 116 | 66.3 | 216 | 140 | 58.7 | 154 | 23 | 13.1 | 215 | 72 | 34.7 |
| 2001-02 | 161 | 113 | 64.9 | 227 | 166 | 65.2 | 161 | 23 | 11.2 | 226 | 51 | 20.7 |
| 2003-04 | 179 | 106 | 52.9 | 218 | 138 | 57.8 | 179 | 32 | 13.4 | 217 | 50 | 20.8 |
| 2005-06 | 171 | 107 | 58.8 | 220 | 151 | 65.8 | 170 | 29 | 12.2 | 220 | 58 | 23.5 |
| 2007-08 | 199 | 127 | 60.2 | 225 | 164 | 67.6 | 198 | 26 | 10.3 | 223 | 71 | 26.5 |
| 2009-10 | 216 | 119 | 49.6 | 246 | 170 | 61.5 | 216 | 35 | 12.8 | 246 | 62 | 21.8 |
| 2011-12 | 220 | 120 | 49.9 | 219 | 140 | 57.1 | 221 | 26 | 11.1 | 218 | 54 | 4.7 |
| 2013-14 | 240 | 139 | 54.7 | 238 | 141 | 53.8 | 240 | 26 | 10.5 | 238 | 54 | 19.5 |
| 2015-16 | 216 | 114 | 41.8 | 225 | 143 | 55.6 | 217 | 18 | 5.5 | 225 | 47 | 17.3 |
| **Age 35-39 years** |  |  |  |  |  |  |  |  |  |  |  |  |
| 1999-00 | 163 | 115 | 65.9 | 182 | 130 | 65.7 | 163 | 27 | 15.3 | 182 | 69 | 38.9 |
| 2001-02 | 185 | 126 | 64.8 | 229 | 163 | 65.5 | 187 | 43 | 20.6 | 228 | 84 | 34.7 |
| 2003-04 | 158 | 117 | 68.4 | 170 | 123 | 69.5 | 158 | 24 | 13.3 | 171 | 51 | 29.1 |
| 2005-06 | 191 | 137 | 65.0 | 176 | 116 | 60.5 | 190 | 36 | 17.1 | 176 | 58 | 30.2 |
| 2007-08 | 234 | 159 | 63.2 | 239 | 183 | 72.2 | 233 | 48 | 15.2 | 239 | 69 | 21.5 |
| 2009-10 | 223 | 139 | 59.0 | 250 | 165 | 57.4 | 222 | 35 | 10.5 | 249 | 80 | 28.6 |
| 2011-12 | 211 | 135 | 59.4 | 210 | 150 | 70.0 | 211 | 30 | 13.3 | 212 | 67 | 28.1 |
| 2013-14 | 195 | 121 | 56.6 | 242 | 177 | 67.3 | 195 | 35 | 15.8 | 241 | 59 | 21.4 |
| 2015-16 | 202 | 131 | 57.6 | 220 | 154 | 63.1 | 204 | 29 | 10.7 | 219 | 50 | 19.7 |
| **Age 40-44 years** |  |  |  |  |  |  |  |  |  |  |  |  |
| 1999-00 | 182 | 129 | 58.1 | 185 | 150 | 74.3 | 181 | 40 | 16.1 | 183 | 69 | 29.4 |
| 2001-02 | 221 | 157 | 61.8 | 209 | 156 | 65.3 | 220 | 41 | 14.3 | 211 | 82 | 36.6 |
| 2003-04 | 184 | 121 | 56.7 | 189 | 147 | 71.3 | 184 | 45 | 19.1 | 187 | 68 | 30.1 |
| 2005-06 | 189 | 133 | 63.0 | 205 | 143 | 64.5 | 189 | 44 | 21.9 | 205 | 63 | 26.1 |
| 2007-08 | 201 | 141 | 59.1 | 223 | 165 | 61.5 | 201 | 50 | 21.3 | 222 | 83 | 31.9 |
| 2009-10 | 244 | 174 | 61.9 | 281 | 214 | 67.1 | 244 | 62 | 20.4 | 281 | 91 | 26.5 |
| 2011-12 | 198 | 150 | 75.2 | 211 | 164 | 75.4 | 198 | 39 | 14.5 | 210 | 87 | 32.3 |
| 2013-14 | 226 | 150 | 60.3 | 282 | 207 | 64.8 | 226 | 31 | 10.6 | 282 | 88 | 30.7 |
| 2015-16 | 182 | 123 | 62.8 | 248 | 171 | 58.5 | 182 | 33 | 13.6 | 248 | 84 | 28.5 |
| **Age 45-49 years** |  |  |  |  |  |  |  |  |  |  |  |  |
| 1999-00 | 130 | 98 | 62.3 | 161 | 118 | 64.0 | 130 | 39 | 25.4 | 161 | 68 | 37.6 |
| 2001-02 | 211 | 164 | 70.6 | 181 | 134 | 67.7 | 212 | 59 | 17.9 | 183 | 73 | 35.1 |
| 2003-04 | 161 | 114 | 67.4 | 168 | 127 | 66.2 | 161 | 44 | 21.1 | 168 | 69 | 34.5 |
| 2005-06 | 192 | 128 | 61.3 | 190 | 128 | 55.2 | 192 | 48 | 20.2 | 190 | 79 | 35.0 |
| 2007-08 | 207 | 159 | 70.5 | 232 | 166 | 64.2 | 205 | 42 | 15.8 | 232 | 99 | 36.6 |
| 2009-10 | 238 | 171 | 61.7 | 267 | 202 | 71.9 | 238 | 57 | 17.6 | 268 | 101 | 34.5 |
| 2011-12 | 190 | 143 | 67.1 | 203 | 157 | 76.5 | 190 | 61 | 28.3 | 202 | 75 | 35.5 |
| 2013-14 | 213 | 149 | 66.3 | 233 | 161 | 61.5 | 210 | 41 | 13.9 | 232 | 79 | 28.8 |
| 2015-16 | 198 | 139 | 53.8 | 227 | 172 | 65.5 | 198 | 42 | 16.7 | 228 | 83 | 25.3 |

^*^Weighted seroprevalence.
