# Supplementary figures and images for: Characterizing herpes simplex virus type 1 and type 2 seroprevalence declines and epidemiological association in the United States
Source: PLoS One. 2019 Jun 6;14(6):e0214151. doi: 10.1371/journal.pone.0214151 (PMC6553692; doi:10.1371/journal.pone.0214151)

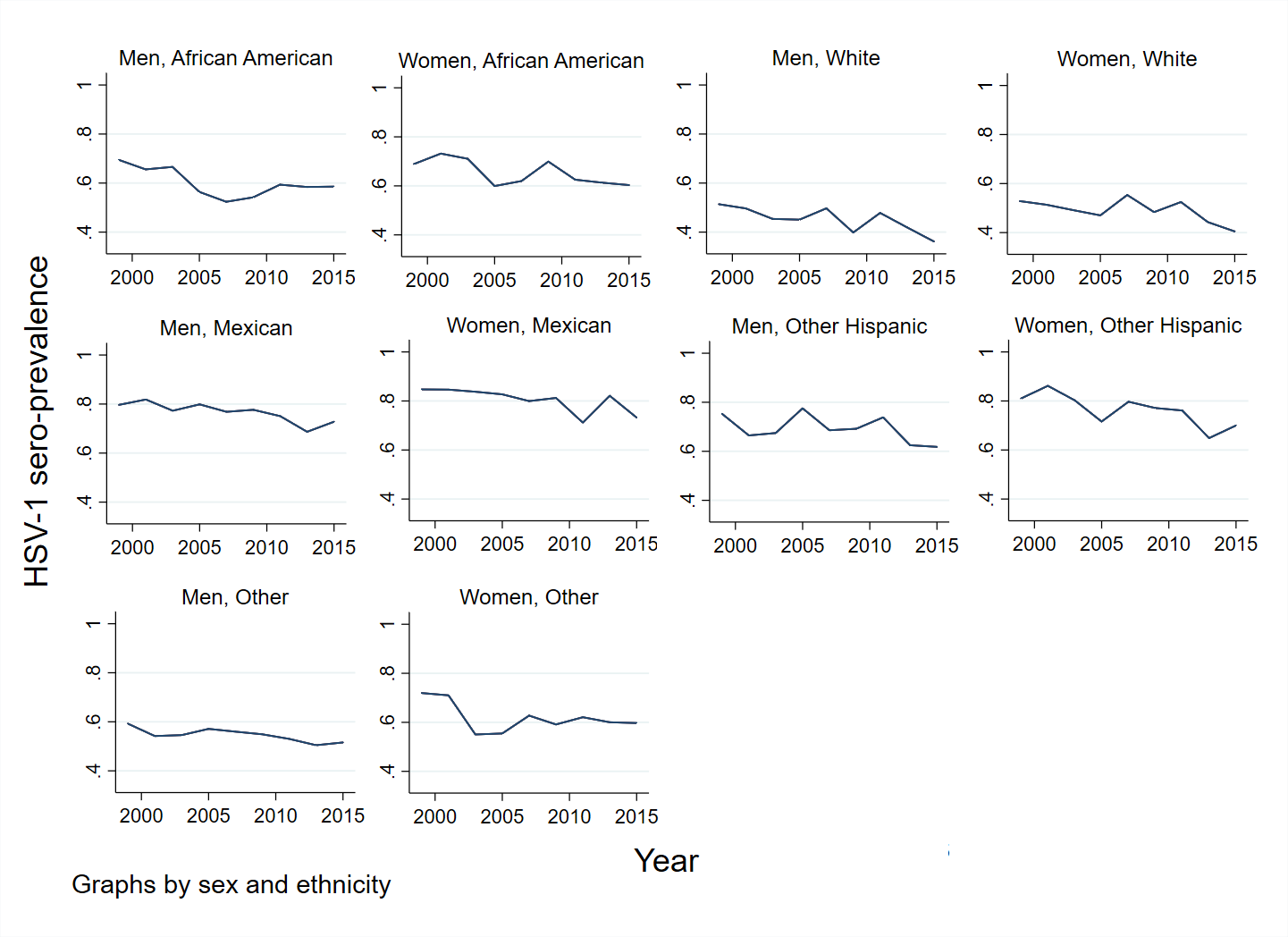

Supplement: S1 Fig — Seroprevalence was standardized with respect to the United States 2010 population (ages 14–49). Interpolating curves were estimated using loess. (TIF) [file pone.0214151.s003.tif]

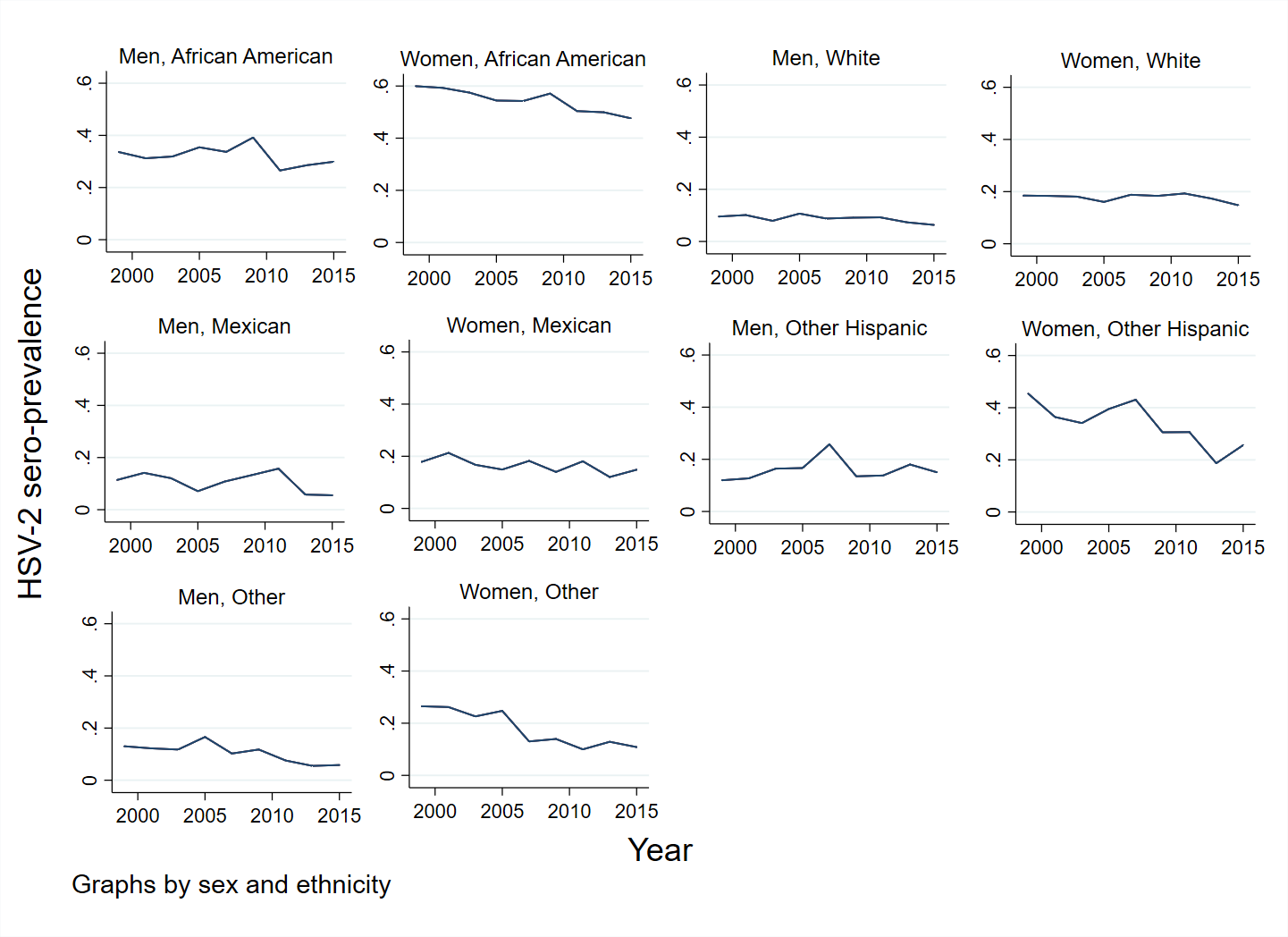

Supplement: S2 Fig — Seroprevalence was standardized with respect to the United States 2010 population (ages 18–49). Interpolating curves were estimated using loess. (TIF) [file pone.0214151.s004.tif]
